# Altered Activation in Cerebellum Contralateral to Unilateral Thalamotomy May Mediate Tremor Suppression in Parkinson’s Disease: A Short-Term Regional Homogeneity fMRI Study

**DOI:** 10.1371/journal.pone.0157562

**Published:** 2016-06-16

**Authors:** Zhi Wen, Jie Zhang, Jielan Li, Jiankun Dai, Fuchun Lin, Guangyao Wu

**Affiliations:** 1 Department of Magnetic Resonance Imaging, Zhongnan Hospital of Wuhan University, Wuhan, Hubei, China; 2 Department of Neurosurgery, Zhongnan Hospital of Wuhan University, Wuhan, Hubei, China; 3 National Center for Magnetic Resonance, Key State Laboratory of Atomic and Molecular Physics, Wuhan Institute of Physics and Mathematics, Chinese Academy of Sciences, Wuhan, Hubei, China; 4 University of Chinese Academy of Sciences, Beijing, China; Institute of Psychology, Chinese Academy of Sciences, CHINA

## Abstract

**Background:**

Ventral intermediate nucleus thalamotomy is an effective treatment for Parkinson’s disease tremor. However, its mechanism is still unclear.

**Purpose:**

We used resting-state fMRI to investigate short-term ReHo changes after unilateral thalamotomy in tremor-dominant PD, and to speculate about its possible mechanism on tremor suppression.

**Methods:**

26 patients and 31 healthy subjects (HS) were recruited. Patients were divided into two groups according to right- (rPD) and left-side (lPD) thalamotomy. Tremor was assessed using the 7-item scale from the Unified Parkinson’s disease rating scale motor score (mUPDRS). Patients were scanned using resting state fMRI after 12h withdrawal of medication, both preoperatively (PD_pre_) and 7- day postoperatively (PD_post_), whereas healthy subjects were scanned once. The regions associated with tremor and altered ReHo due to thalamic ablation were examined.

**Results:**

The impact of unilateral VIM thalamotomy was characterized in the frontal, parietal, temporal regions, basal ganglia, thalamus, and cerebellum. Compared with PD_pre_, significantly reduced ReHo was found in the left cerebellum in patients with rPD_post_, and slightly decreased ReHo in the cerebellum vermis in patients with lPD_post_, which was significantly higher than HS. We demonstrated a positive correlation between the ReHo values in the cerebellum (in rPD, peak coordinate [-12, -54, -21], R = 0.64, *P* = 0.0025, and peak coordinate [-9, -54, -18], R = 0.71, *P* = 0.0025; in lPD, peak coordinate [3, -45, -15], R = 0.71, *P* = 0.004) in the pre-surgical condition, changes of ReHo induced by thalamotomy (in rPD, R = 0.63, *P* = 0.021, R = 0.6, *P* = 0.009; in lPD, R = 0.58, *P* = 0.028) and tremor scores contralateral to the surgical side, respectively.

**Conclusion:**

The specific area that may be associated with PD tremor and altered ReHo due to thalamic ablation is the cerebellum. The neural basis underlying thalamotomy is complex; cerebellum involvement is far beyond cerebello-thalamic tract breakage.

## Introduction

Parkinson's disease (PD) is a progressive neurodegenerative disease in the elderly. Almost all patients experience tremor at rest in the disease process [1]. The depletion of dopamine in the substantia nigra is the predominant neurochemical hallmark of PD, and as the disease progresses, it leads to dysfunction of striatal-thalamo-cortical circuits, involving non-dopaminergic brain areas, resulting in motor and non-motor symptoms [2]. Although this hypothesis serves as a critical reason for motor dysfunctions in PD, i.e. akinesia and rigidity, conversely, it fails to explain tremor [3]. Evidence suggests that loss of dopaminergic neurons in the basal ganglia correlates consistently with clinical ratings of akinesia and rigidity, rather than tremor [4]. Indeed, clinical studies provide evidence that the level of tremor severity is independent of the amount of dopamine deficiency [5] and is often refractory to dopamine replacement treatment [6]. Thus, tremor might be a unique symptom in PD.

Evidence that the thalamus is associated with PD tremor originates from the impact of stereotactic thalamotomy [7]. Neuromodulation in the form of stereotactic thalamotomy is an approved therapy to alleviate intractable tremor in developing countries [8]. Previous literature review has demonstrated that tremor suppression is observed immediately after creation of the lesion, and is still effective postoperatively [9]. Thalamic ablation, which targets the posterior ventral lateral (VLp) or ventral intermediate (VIM) nucleus, is able to suppress PD tremor [9]. The VIM nucleus is a subdivision of the motor thalamus, according to Hassler’s classification, containing complex overlaying distinct tracts, as cerebello-thalamic excitatory afferents terminate here and project to motor cortical areas [10]. This indicates that the cerebello-thalamo-cortical circuits may be involved in tremor generation. However, it remains elusive how dysfunction of striatal-thalamo-cortical circuits in PD can drive the distinct cerebello-thalamo-cortical circuits into generating resting tremor; and the physiological mechanism of VIM thalamotomy that leads to the tremor suppression in PD.

In the last decade, the development of resting state functional magnetic resonance imaging (rs-fMRI) has provided researchers with a non-invasive *in-vivo* tool to explore human brain function by using a blood oxygen level dependent (BOLD) effect at rest. The regional homogeneity (ReHo), which applies Kendall’s coefficient concordance (KCC) [11], measures the similarity of time series of a voxel and its given cluster of neighbor voxels [12]. It is a data-driven method of rsfMRI without a priori knowledge of the experimental design. The test–retest reliability for ReHo to reveal the regional synchronization of spontaneous brain activity has also been validated [13, 14]. This method has been successfully used to investigate the functional modulations in the resting state in patients with PD and motor subtypes [15]. Prior rs-fMRI study has revealed abnormal neural activity concerning PD tremor in several regions, namely the thalamus, basal ganglia, cortex, and cerebellum [3]. Indeed, many functional imaging studies have discussed the activation differences between tremor-dominant (TD) and non-tremor subtypes [3, 4, 16]. For example, Zhang et al. [4] illustrated that TD PD had increased ReHo in the left thalamus, primary motor cortex (M1), and cerebellum, whereas decreased ReHo in the left primary sensorimotor cortex (SM1) and putamen, when compared with akinetic-rigid PD. It suggests that PD tremor is associated with dysfunction of striatal-thalamo-cortical and cerebello-thalamo-cortical circuits [17]. However, there has been no published rs-fMRI study on thalamic ablation in TD PD, because the surgical changes relative to tremor suppression may be confounded by the non-specific effects derived from tissue lesions.

Based on previous studies, we hypothesized that clinical suppression of PD tremor due to VIM thalamotomy may be reflected by the ReHo alterations mainly in the cortex, basal ganglia, and cerebellum. During this study, we: (1) clarified the surgical impact by examining the ReHo differences in asymmetric TD PD preoperatively versus 7 –day postoperatively (PD_post_ versus PD_pre_); (2) examined the ReHo differences of TD PD from healthy subjects (HS) (PD_pre_ versus HS) and association with tremor scores; (3) speculated about the possible brain areas that mediate the surgical impact on tremor suppression.

## Materials and Methods

### Ethics statement

This study was approved by Zhongnan Hospital of Wuhan University Ethics Committee (Ethics No. [2014003]). All study procedures were in accordance with the Declaration of Helsinki. For the purpose of the study, all the patients were required to stop taking dopamine medication for at least 12 hours. All patients were well informed of the risks, such as orthostatic hypotension, paroxysmal nocturnal dyspnea, and urinary disorders. All participants gave written consent before the study.

### Subjects

31 patients with TD PD (12 females, 19 males, age 60.1 ± 8.17 years) who underwent unilateral VIM thalamotomy were recruited at the Department of Neurosurgery in Zhongnan Hospital of Wuhan University from March 2014 to August 2015. An additional 31 healthy subjects (16 females, 15 males, age 59.6 ± 7.65 years) were also measured. All patients were diagnosed based on UK Brain Bank Criteria for idiopathic PD [12] and demonstrated an asymmetric resting tremor during physical examination. The exclusion criteria were: (1) cognitive dysfunction measured as a Mini-Mental State Exam (MMSE) [13] score < 26; (2) history of brain trauma or surgery; (3) history of neurological and neuropsychiatric diseases; and (4) clinically silent lesion evident on conventional MRI. Clinical descriptive information for healthy subjects and patients with Parkinson’s disease is shown in Table 1.

**Table 1:** Clinical details of healthy subjects and patients with Parkinson’s disease.

	HS (*n* = 31)	rPD (*n* = 12)	lPD (*n* = 14)	*P*
Age (y)	59.6 ± 7.65	60.8 ± 7.02	61.4 ± 7.77	0.87^a^
Gender (M/F)	15/16	8/4	6/8	0.44^b^
Handedness	Right	Right	Right	
MMSE	>26	>26	>26	
Disease/ Tremor duration (y)	-	5 (1–17)	5.5 (1–14)	0.74^c^
mUPDRS	-	28.9 ± 10.9	26.4 ± 15.6	0.47^c^
Tremor score contralateral to the surgical side	-	2.92 ± 0.79	3 ± 0.68	0.76^c^

^a^ ANOVA;

^b^ Pearson Chi-square test;

^c^ Mann-Whitney U test.

mUPDRS, Unified Parkinson’s Disease Rating Scale motor score; HS, healthy subjects; rPD, patients receiving right-side VIM thalamotomy; lPD, patients receiving left-side VIM thalamotomy; F, female; M, male.

Patients with TD PD were assessed by Unified Parkinson’s Disease Rating Scale motor score (mUPDRS) [14] preoperatively and 7-day postoperatively, after dopaminergic medication had been withdrawn for more than 12 hours (OFF state). Tremor was assessed using a 7-item scale that included mUPDRS items 20 and 21 [18]: resting tremor of the head and the right and left limbs (five items), as well as postural tremor of each hand (two items). Each item was rated 0–4 with 0 representing absence of the symptom and 4 indicating significant presence of the symptom. The tremor scale was calculated for each side, and then the ratio (right: left score) determined. Based on this method, predominantly left-side affected subjects had a ratio < 1, whereas right-side affected subjects had a ratio > 1. The surgical side was determined to suppress the contralateral tremor-dominant symptom and patients were then further divided into two groups: patients with right-side VIM thalamotomy (rPD) and patients with left-side VIM thalamotomy (lPD). The surgical procedure is mentioned in the S1 Text. There were no complications such as hemorrhage, edema, or intracranial infection, identified by conventional MRI, either immediately or 7-day postoperatively. We sent a questionnaire to PD patients via mail to enquire about the ablation effect at 3 months after surgery. All patients had diminished tremor and the tremor score was equal to 0, which guaranteed the impact of tremor alleviation.

### Functional MRI acquisition

During the whole study procedure, patients were accompanied by at least one experienced neurosurgeon. The rs-fMRI was performed immediately after clinical assessments with a 3.0 T MRI scanner (Magneto Trio, Siemens Erlangen, Germany), using a gradient-echo echo-planar sequence sensitive to BOLD contrast with the following parameters: TR/TE = 2000/30 ms, flip angle = 90°, matrix size = 64 × 64, FOV = 240 mm × 240 mm, slice thickness = 5 mm with no gap. Each fMRI scan lasted 7 minutes, and 30 axial slices were collected. A standard 8-channel head coil was used with foam padding to restrict head motion. During rs-fMRI, participants were instructed to keep their eyes closed, to keep still, and to clear their minds of thought.

Anatomic imaging was acquired using T1-3D MPRAGE with the following parameters: TR/TE/TI = 1900/2.1/900 ms, flip angle = 9°, matrix size = 256 × 256, FOV = 240 mm × 240 mm, slice thickness = 1 mm with no gap. T2- weighted images were also obtained in every subject to detect clinically silent lesions.

### Data analysis and statistical analysis

Image data were analyzed using DPARSF [19] and REST [20] based on Matlab 2010a (Mathworks, Natick, Massachusetts, USA). The data for each fMRI scan contained 210 time points. The first 10 time points of fMRI data were discarded because of the instability of the transient signal and to allow subjects to get used to the scanning noise. The image pre-processing procedure included slice timing, head motion correction, co-registration to individuals’ T1 image, and spatial normalization to the Montreal Neurological Institute (MNI) space. No subjects exceeded the head motion threshold of 3 mm of displacement or 3 degrees of rotation.

Linear drift was removed. Low-frequency drift and high-frequency physiological noise were removed by using a temporal filter (0.01 Hz < f < 0.08 Hz). Kendall’s coefficient concordance (KCC) [11] was calculated to generate individual ReHo maps between the time series of a given voxel and those of its 26 nearest neighbors. For standardization purposes, each individual ReHo map was divided by its own mean ReHo within the mask that was extracted from the intracranial voxels. The head motion parameter [21], as well as white matter and CSF signals, were regressed out to remove confounding artifacts. Spatial smoothing was then applied to the ReHo maps (full width at half maximum, FWHM = 4 mm), to reduce noise and residual differences in gyral anatomy.

Voxel-by-voxel based comparisons of ReHo differences were performed between PD groups in the pre- and post-surgical conditions using Student’s paired *t*-test in REST software (PD_post_ versus PD_pre_). Comparisons of ReHo differences were also performed between the healthy subjects and PD patients in the pre-surgical condition (PD_pre_ versus HS), and between the healthy subjects and PD patients in the post-surgical condition (PD_post_ versus HS), using a two-sample *t*-test with age and gender as covariates. The significance of group differences was set at *P* < 0.005, cluster size ≥ 23 voxels, corresponding to AlphaSim corrected *P* < 0.05 (http://afni.nimh.nih.gov/pub/dist/doc/manual/AlphaSim.pdf). In addition, voxel-by-voxel correlation coefficients were also calculated between ReHo of PD_pre_ and tremor score contralateral to the surgery side (*P* < 0.005, uncorrected, cluster size ≥ 8 voxels).

The effect of surgery on PD tremor was explored. Firstly, the overlapping brain areas in PD_pre_, where ReHo values were altered relative to HS and correlated with tremor scores, were identified. Secondly, the overlapping brain areas in PD_post_, where there was a ΔReHo relative to PD_pre_ and which correlated with Δtremor scores, were identified. Spherical ROI was drawn using the peak coordinate as the origin and radius = 6 mm. The mean of the ReHo was extracted among HS, PD_pre_ and PD_post_. One-way ANOVA and post-hoc analysis were performed using SPSS 19.0 (Chicago, IL, USA) (*P* < 0.05, Bonferroni corrected). The change of ReHo between the pre- and post-surgical condition (ΔReHo) was calculated as the correlation coefficient with tremor score (*P* < 0.05, Spearman corrected).

## Results

### Clinical assessments

5 patients were excluded due to their intolerance of the second MRI scanning. Their MRI data was not collected completely. Finally, 26 patients with PD and 31 HS were included in subsequent data analysis. No difference was found in either age or gender among HS, rPD and lPD (*P* > 0.05). There were no significant differences in the disease duration, total mUPDRS, and tremor score contralateral to the surgical side, between rPD and lPD (*P* > 0.05). Moreover, compared with PD_pre_, total, the mUPDRS and tremor score of TD PD contralateral to the surgical side reduced significantly after unilateral VIM thalamotomy (*P* < 0.005) (Table 2).

**Table 2:** Severity of disease in patients with PD, pre- and postoperatively.

Severity of disease	Pre	Post	*P*
mUPDRS			
rPD	28.9 ± 10.9	11.5 ± 6	0.002^a^
lPD	26.4 ± 15.6	10.6 ± 8.3	0.001^a^
Tremor score contralateral to the surgical side			
rPD	2.92 ± 0.79	0	0.001^a^
lPD	3 ± 0.68	0	0.002^a^

^a^Wilcoxon Signed Ranks test.

mUPDRS, Unified Parkinson’s Disease Rating Scale motor score; rPD, patients receiving right-side VIM thalamotomy; lPD, patients receiving left-side VIM thalamotomy.

### Changes in ReHo of brain areas between the pre- and post-surgical conditions in PD (PD_post_ versus PD_pre_)

**Fig 1:**
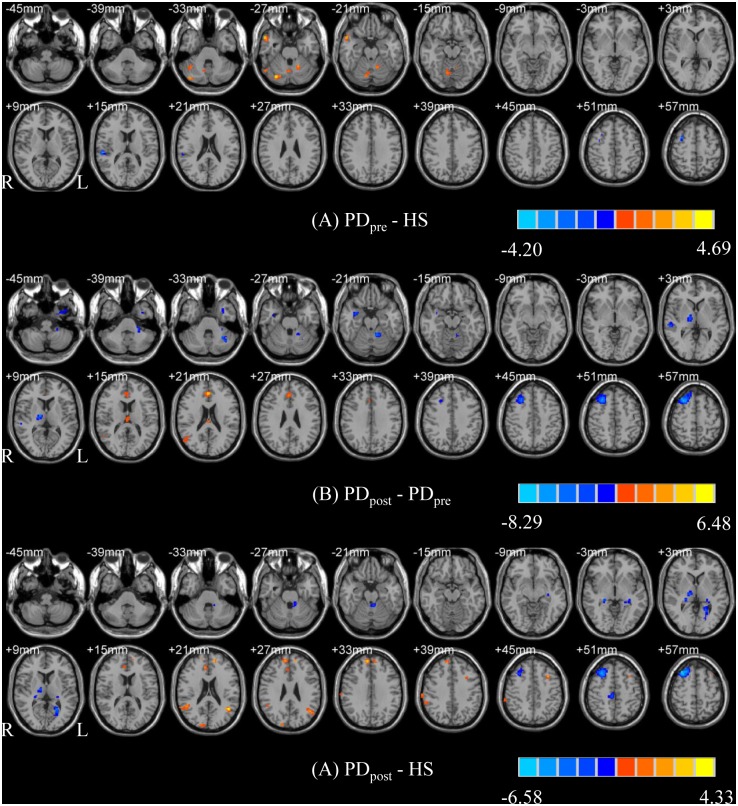
T- statistics maps of rPD_pre_ versus HS (A), rPD_post_−rPD_pre_ (B), and rPD_post_−HS (C). T-score bar: hot and cold colors indicate ReHo increases and decreases, respectively. rPD: patients receiving right-side VIM thalamotomy.

**Fig 2:**
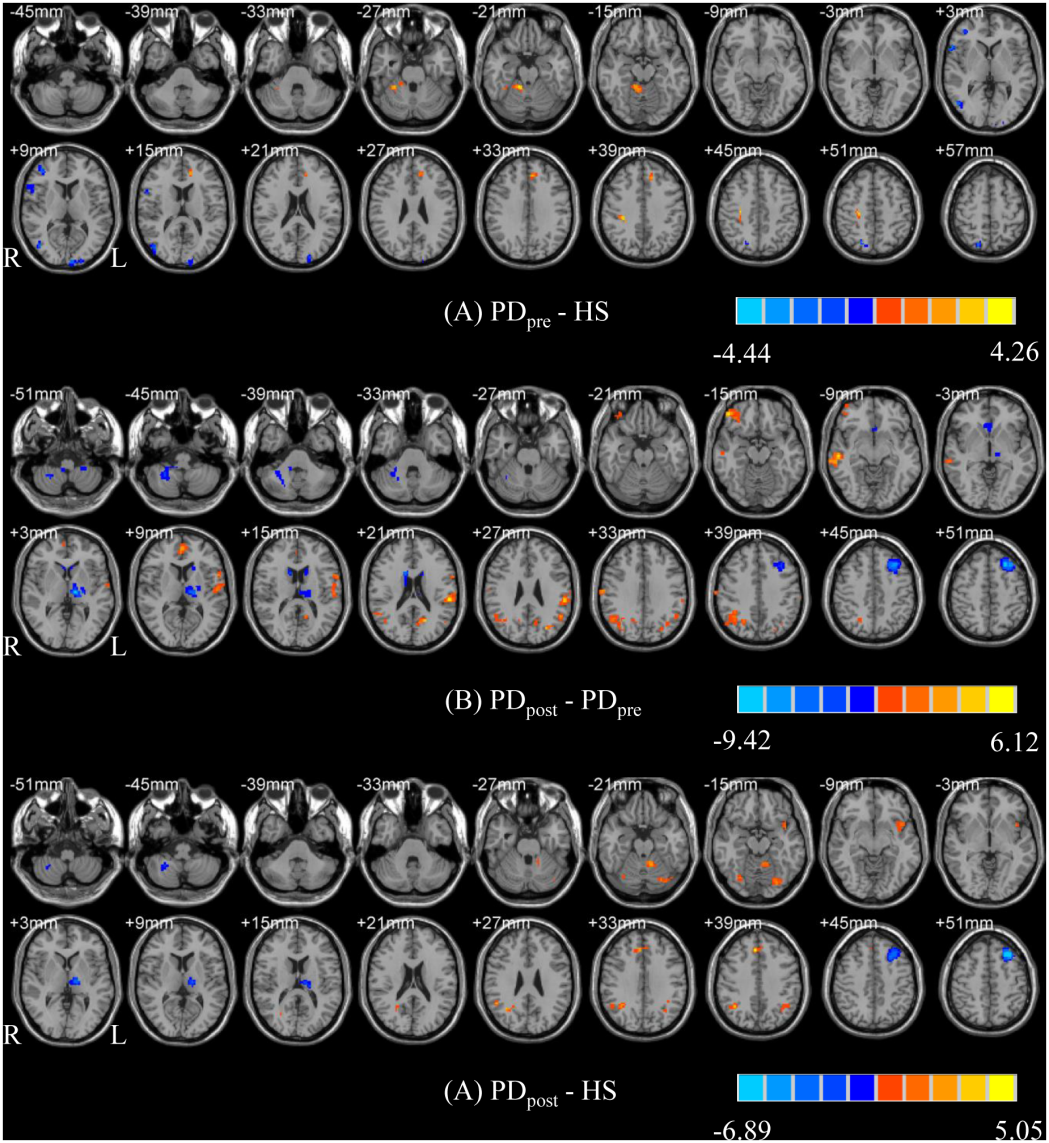
T- statistics maps of lPD_pre_ versus HS (A), lPD_post_−lPD_pre_ (B), and lPD_post_−HS (C). T-score bar: hot and cold colors indicate ReHo increases and decreases, respectively. lPD: patients receiving left-side VIM thalamotomy.

Compared with rPD_pre_, the rPD_post_ showed increased ReHo in the right superior frontal gyrus (SFG), right precentral gyrus, right middle temporal gyrus (MTG), right anterior cingulum (ACC), and left thalamus; and decreased ReHo in the right middle frontal gyrus (MFG), bilateral superior temporal gyri (STG), right hippocampus, right thalamus, and left cerebellum. The details of brain areas with significant ReHo differences are listed in S1 Table. In lPD_post_, higher ReHo was detected in the left MFG, left SFG, left inferior frontal gyrus (IFG), left inferior parietal lobule (IPL), bilateral postcentral gyrus, right MTG, bilateral angular gyrus, left middle occipital gyrus (MOG), right superior occipital gyrus (SOG), left ACC, and left cuneus; whereas lower ReHo was found in the left MFG, left thalamus, bilateral caudate, right ACC, and bilateral cerebellum. The details of brain areas with significant ReHo differences are listed in S1 Table. The PD_post_−PD_pre_ contrast images are shown in Figs 1B and 2B.

### Changes in ReHo of brain areas for PD patients relative to healthy subjects

Compared with HS, the rPD_pre_ had increased ReHo in the right MTG and bilateral cerebellum, as well as decreased ReHo in the right MFG and right STG. For the lPD_pre_, the authors observed higher ReHo in the left SFG, right postcentral gyrus, left ACC, and right cerebellum, co-varying with lower ReHo in the right MFG, right IFG, right superior parietal lobule (SPL), right precentral gyrus, right MTG, left SOG and bilateral MOG. The details of brain areas with significant ReHo differences are listed in S2 Table. The PD_pre_−HS contrast images are shown in Figs 1A and 2A.

Compared with HS, the rPD_post_ revealed significantly increased ReHo in the bilateral SFG, left MFG, right STG, bilateral MTG, right IPL, right SOG and right IPL, and reduced ReHo in the right MFG, right paracentral lobule, left hippocampus, right thalamus, left calcarine and left cerebellum. The lPD_post_ group exhibited significantly increased ReHo in the right SFG, left STG, left IPL, bilateral angular gyrus, bilateral fusiform gyrus, left insula, and left cerebellum, and decreased ReHo in the left MFG, left thalamus, and right cerebellum. The details of brain areas with significant ReHo differences are listed in S3 Table. The PD_post_−HS contrast images are shown in Figs 1C and 2C.

### Association between ReHo and tremor scores contralateral to the surgical side

A significant negative correlation was shown between tremor scores contralateral to the surgical side and ReHo in the right caudate in rPD_pre_ and right precuneus in lPD_pre_, accompanied by a positive correlation in the left IPL, left cerebellum anterior lobe in rPD_pre_ and cerebellum vermis in lPD_pre_. The details of brain areas correlated with tremor score are listed in S4 Table.

The ΔReHo in the left MFG, right postcentral gyrus, right IOG in rPD, as well as right SPL, right precuneus, right postcentral gyrus, and left MOG in lPD, were negatively correlated with tremor score contralateral to the surgical side. Conversely, ΔReHo in the cerebellum culmen, left cerebellum posterior lobe in rPD, as well as right cerebellum posterior lobe in lPD, were positively correlated with tremor score.

### The effect of surgery on brain areas relative to PD tremor

**Fig 3:**
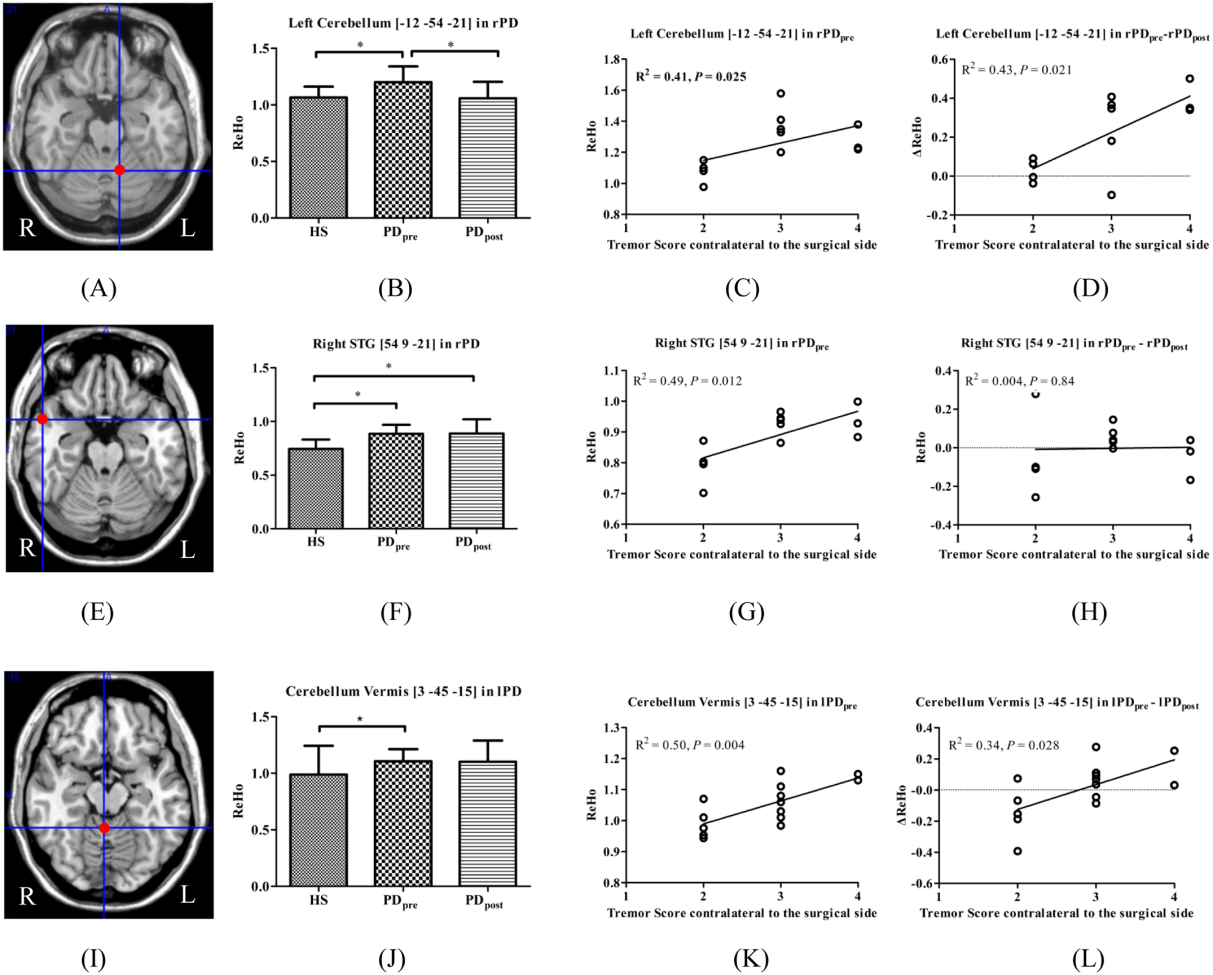
Brain areas which demonstrated altered ReHo relative to HS and correlated with tremor. (A) left cerebellum_4_5 in rPD; (E) right STG in rPD; (I) cerebellum vermis_3 in lPD; (B, F, J) ReHo among HS, PD_pre_, and PD_post_; (C, G, K) ReHo in PD_pre_ correlated with tremor; (D, H, L) ΔReHo correlated with tremor.

There were overlapping regions of the altered ReHo values relative to HS that correlated with tremor scores in PD. In rPD, these were located in the left cerebellum anterior lobe (peak coordinate [-12, -54, -21]) and right STG (peak coordinate [54, 9, -21]), but in the cerebellum vermis (peak coordinate [3, -45, -15]) in lPD. Compared with HS, significantly increased ReHo was found in the left cerebellum in rPD_pre_, which was significantly decreased in rPD_post_. The cerebellum vermis in lPD manifested a similar change pattern, but was not statistically significant. Moreover, the right STG showed significantly higher ReHo in rPD_pre_ relative to HS, which was even higher in rPD_post_ (Fig 3).

**Fig 4:**
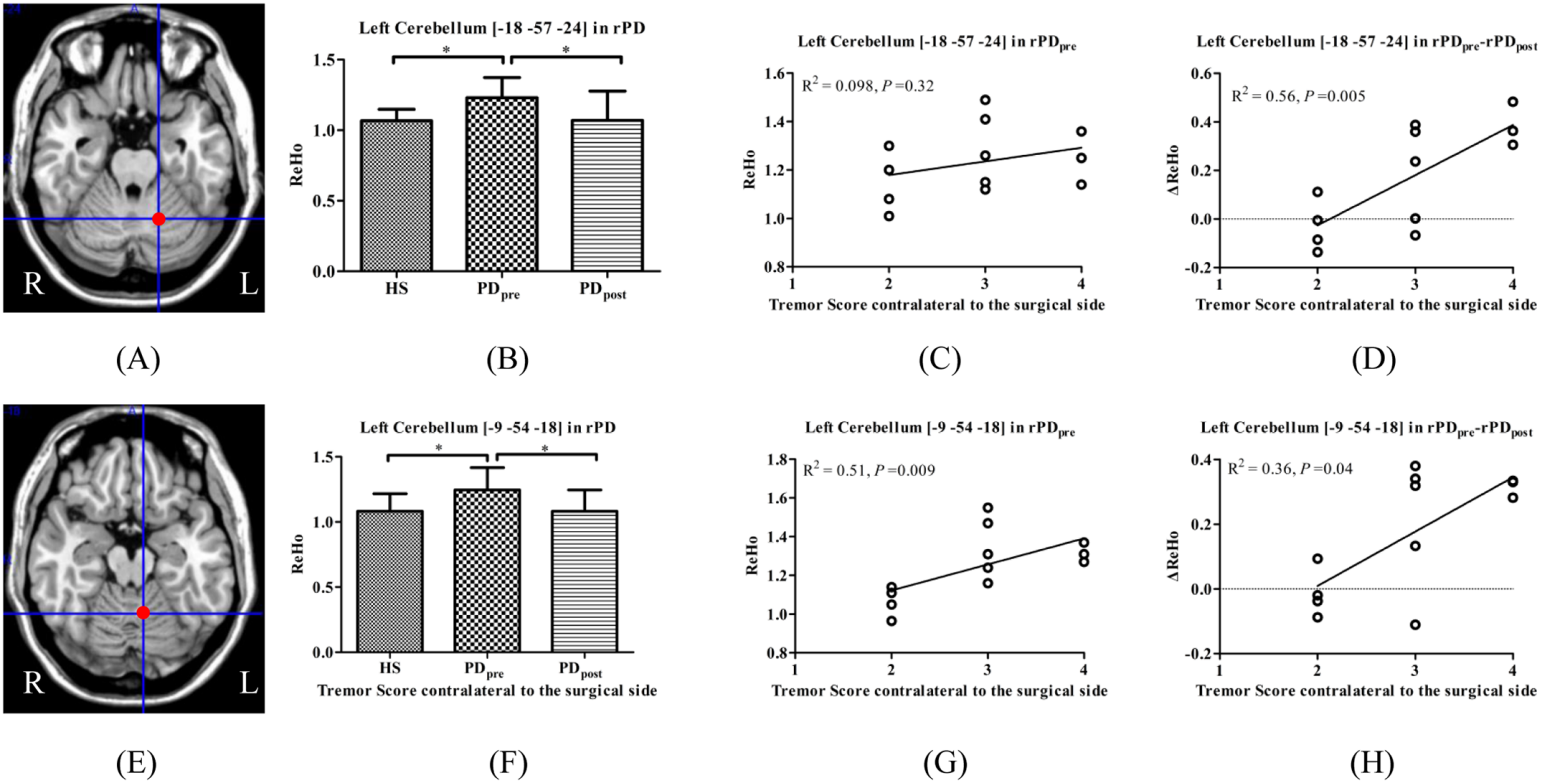
Brain areas which showed significant ΔReHo in PD_post_ versus PD_pre_ and correlated with tremor. (A) left cerebellum_6 in rPD; (E) left cerebellum_4_5 in rPD; (B, F) ReHo among HS, PD_pre_, and PD_post_; (C, G) ReHo in PD_pre_ correlated with tremor; (D, H) ΔReHo correlated with tremor.

Moreover, the left cerebellum (peak coordinate [-18, -57, -24] and [-9, -54, -18]) in rPD are identified as the brain areas of significant ΔReHo due to surgery, and correlated with Δtremor scores. Compared with HS, the left cerebellum showed significantly increased ReHo in rPD_pre_, which was significantly decreased in rPD_post_ (Fig 4).

## Discussion

In this study, the ReHo method was used to explore short-term (7 days) brain functional changes after unilateral VIM thalamotomy and to investigate its efficacy on PD tremor suppression. Our results reveal that the impact of unilateral VIM thalamotomy is characterized in the frontal, parietal, temporal, occipital regions, basal ganglia, thalamus, and cerebellum. The specific area that is associated with PD tremor and altered ReHo due to thalamic ablation may be the cerebellum. However, the change in pattern does not imply conformity in rPD and lPD.

### Altered ReHo in the cerebellum

The tremor and cerebellar involvement are discussed in PD. It is suggested that a secondary dysfunction in the cerebello-thalamo-cortical (CTC) circuit is responsible for the occurrence of resting tremor, involving the VIM of thalamus, motor cortex, and cerebellum [3]. Systematic review suggested that the role of the cerebellum in PD tremor is a combination of pathological and compensatory effects [10].

Our study reports multiple clusters in the cerebellum in PD_pre_ where ReHo increased significantly relative to HS, such as bilateral cerebellum_6, vermis_3, and right cerebellum_crust1 in rPD, as well as right cerebellum_6 in lPD. Previous studies have documented that regional hypermetabolism in the basal ganglia, cerebellum, and primary motor cortex [22] and functional connectivity increase within the cerebello-thalamic circuit [3] in TD PD. Zhang et al. [23] suggest common “overheated” functional activities in TD PD, which is in accordance with our results. Our study provides evidence that the hyperactivation in the cerebellum is associated with tremor.

The VIM nucleus of the thalamus is one of the targets for ablation and stimulation to suppress PD tremor [9]. Cerebello-thalamic excitatory afferents terminate here and project to motor cortical areas [10]. Clinical studies which interfered in the cerebello-thalamic circuit [24] could effectively suppress resting tremor. A review describes it in terms of functional normalization [10]. Compared with PD_pre_, our study showed multiple clusters in the cerebellum where ReHo reduced significantly in PD_post_, such as left cerebellum_4_5, left cerebellum_crust1, left cerebellum_6, and left cerebellum_10 in rPD and right cerebellum_6, bilateral cerebellum_8 in lPD. The decreased ReHo in the cerebellum after the VIM ablation supports a functional normalization. Of note, with the administration of thalamotomy, the ReHo in the cerebellum of the PD patients reduced, but was still higher than that of healthy subjects.

In order to explore the neural basis of ReHo alteration in the cerebellum due to thalamic ablation, we distinguished its effect on PD tremor from breakage of cerebello-thalamic fibers. Hoshi et al. [25] applied transneuronal transport of rabies virus in macaques, indicating that the outflow pathway was from deep cerebellar nuclei to thalamus via dentate neurons. In our study, we roughly observed that the ReHo in the same right cerebellum_8 decreased in lPD_post_ relative to lPD_pre_ and HS. There is a high probability that the decreased ReHo here after surgery is due to the lesion of the cerebello-thalamic fibers. This finding highlights the important role of cerebello-thalamic circuits in understanding PD tremor, and indicates that tractography studies should be drawn in future.

We obtained intersections at different contrasts to discriminate the possible brain areas that may mediate tremor alleviation induced by surgery. We identified that in the left cerebellum_4_5 (peak coordinate [-12, -54, -21]) and vermis_3 (peak coordinate [3, -45, -15]), ReHo values relative to HS and were positively correlated with tremor scores in rPD and lPD, respectively. Afterwards, although there was slightly reduced amplitude of ReHo in vermis_3 in lPD after surgery, which was not statistically significant, ΔReHo was positively correlated with Δtremor scores in rPD and lPD. The left cerebellum_6 (peak coordinate [-18, -57, -24]) and left cerebellum_4_5 (peak coordinate [-9, -54, -18]) revealed significant ΔReHo due to surgery, and positively correlated with Δtremor scores in rPD instead of lPD. However, only the ReHo of the left cerebellum_4_5 (peak coordinate [-9, -54, -18]) in rPD_pre_ was positively correlated with tremor scores. Together, these findings favor the view of a critical role for the cerebellum in the generation of Parkinsonian tremor. The left cerebellum anterior lobe may mediate tremor alleviation caused by right-sided thalamotomy. Compared with right-sided surgery, a complex mechanism may underlie ReHo changes in the right cerebellum after left-sided thalamotomy in PD.

### Altered ReHo in the cortical areas and basal ganglia

According to the pathological theory of Braak et al. [26], PD involves the functional alteration of whole brain networks rather than localized neurodegeneration. Tessitore et al. [27] reported reduced resting state functional connectivity of right MTG and bilateral IPL within the default mode network (DMN) in PD, compared with HS. Besides DMN, specific changes have been found in the fronto-parietal, sensorimotor, visual and auditory networks [16]. In our study, altered ReHo was found in the right MFG, right MTG and right STG in rPD, as well as in the frontal (i.e. left SFG, right IFG, right MFG, right precentral gyrus) cortex, parietal (i.e. right postcentral gyrus, right SPL), right MTG, occipital (i.e. left SOG, bilateral MOG) cortex, and ACC in lPD, relative to HS, which is coherent with previous studies [28, 29].

In addition, the cerebello-thalamo-cortical (i.e. cerebellum, thalamus, SM1, and M1) and striatal-thalamo-cortical (i.e. striatum, thalamus, SMA, SM1, M1) circuits are distinct, but with considerable overlapping in the motor cortical areas. Fukuda et al. [30] performed PET study combined with triaxial accelerometry to examine the effect of thalamic stimulation on PD tremor. The result showed that the activation in SM1 was associated with tremor, indicating that the interference with cerebello-thalamo-cortical circuits (VIM of the thalamus) may modulate the striatal-thalamo-cortical circuits at the cortical level. Similarly, we detected the ReHo increases in the bilateral postcentral gyri and decreases in the bilateral caudate in lPD_post_ relative to lPD_pre_, which is coherent with previous studies.

### Altered ReHo in PD_post_ versus HS

The PD_post_ versus HS contrast comparison is complex in this study. Ideally, there are no differences between PD_post_ and HS if patients with PD have fully recovered, as they are exactly the same as HS. In fact, our study found ReHo changes in the frontal, parietal, limbic, and insular cortices. There are several confounding factors which need to be taken into account, such as the surgical brain tissue lesion, surrounding edema and inflammation, and more importantly, the surgical impact on PD tremor (positive aspect) and side effects (negative aspect). Unfortunately, we could not identify the specific neuro-mechanism underlying the PD_post_ relative to HS based on current data.

## Limitations

There are two main issues that need to be considered in future. Firstly, PD is a common neurodegenerative disease with a high degree of heterogeneity [31]. However, the current sample size is relatively small, which limits the power of the study. Secondly, although our study identified cerebellum involvement, it did not clarify the possible mechanism underlying tremor generation and alleviation by thalamic ablation. Indeed, our conclusion is drawn based on the differences of 7 days; a longitudinal study is needed to provide insights on the long-term impact of thalamic ablation on PD tremor.

## Conclusion

This study provides evidence that the impact of thalamotomy is observed in the cortical, subcortical regions and cerebellum. Altered activation in the cerebellum may mediate generation and alleviation of PD tremor. However, the role of the cerebellum may be complex. Cerebellum involvement is far beyond cerebello-thalamic tract breakage. Further studies are needed with a large sample size, longitudinal observation, and multi-modality imaging analysis.

## Supporting Information

S1 Text Surgical procedure of VIM thalamotomy. (DOCX)

S1 Table ReHo differences of PD patients between the pre- and post-surgical conditions. (DOCX)

S2 Table ReHo differences between HS and PD patients in the pre-surgical condition. (DOCX)

S3 Table ReHo differences between HS and PD patients in the post-surgical condition. (DOCX)

S4 Table Brain areas showing significant correlations between ReHo in PD_pre_ and tremor score contralateral to surgical side. (DOCX)
