# Supplementary material for: Altered Activation in Cerebellum Contralateral to Unilateral Thalamotomy May Mediate Tremor Suppression in Parkinson’s Disease: A Short-Term Regional Homogeneity fMRI Study
Source: PLoS One. 2016 Jun 16;11(6):e0157562. doi: 10.1371/journal.pone.0157562 (PMC4910974; doi:10.1371/journal.pone.0157562)
Supplement: S1 Text — (DOCX) [file pone.0157562.s001.docx]

**Surgical procedure of VIM thalamotomy**

After attachment of the Leksell G (Elekta Inc., Sweden) stereotaxic frame, 3D-T1WI MR imaging of the brain was undertaken to determine the three-dimensional coordinates of the anterior and posterior commissures (AC, PC). Generally, the VIM nucleus was located 4-5 mm posterior to the mid AC- PC plane, 13.5-15 mm lateral to the midline, and 0-1 mm above the level of the intercommissural plane. For those patients with a dilated third ventricle, 1-2 mm was added to the lateral dimension [[1](#_ENREF_1)]. A microelectrode was inserted into the target under local anesthetic. The target was confirmed by noting the responses to electrical stimulation through the electrode. Lesions were then made by heating the electrode tip to between 70°C and 76°C. Postoperative confirmation of lesion location was obtained in all patients.

## Reference

1. Miyagishima T, Takahashi A, Kikuchi S, Watanabe K, Hirato M, et al. (2007) Effect of ventralis intermedius thalamotomy on the area in the sensorimotor cortex activated by passive hand movements: fMR imaging study. Stereotact Funct Neurosurg 85: 225-234.
