# Supplementary material for: Altered Activation in Cerebellum Contralateral to Unilateral Thalamotomy May Mediate Tremor Suppression in Parkinson’s Disease: A Short-Term Regional Homogeneity fMRI Study
Source: PLoS One. 2016 Jun 16;11(6):e0157562. doi: 10.1371/journal.pone.0157562 (PMC4910974; doi:10.1371/journal.pone.0157562)
Supplement: S1 Table — (DOCX) [file pone.0157562.s002.docx]

**ReHo differences of PD patients between the pre- and post-surgical conditions**

| Region | No. of voxel | Peak MNI coordinate | | | Peak T intensity |
| --- | --- | --- | --- | --- | --- |
|  |  | x | y | z |  |
| **rPD_post_ > rPD_pre_** | | | | | |
| Frontal_Sup_R | 48 | 18 | -3 | 75 | 7.33 |
| Precental_R |  | 21 | -15 | 69 | 3.89 |
| Cingulum_Ant_R | 72 | 3 | 36 | 21 | 7.16 |
| Thalamus_L | 34 | -3 | -6 | 12 | 3.78 |
| Temporal_Mid_R | 33 | 54 | -63 | 18 | 4.71 |
| **rPD_post_ < rPD_pre_** | | | | | |
| Frontal_Mid_R | 335 | 30 | 21 | 54 | -9.58 |
| Thalamus_R | 54 | 15 | -12 | 9 | -7.97 |
| Cerebellum_4_5_L | 93 | -12 | -48 | -21 | -6.74 |
| Cerebellum_Crust1_L |  | -33 | -54 | -33 | -5.72 |
| Cerebellum_6_L |  | -24 | -50 | -24 | -4.31 |
| Hippocampus_R | 26 | 36 | -3 | -21 | -6.15 |
| Cerebellum_10_L | 23 | -24 | -36 | -42 | -5.48 |
| Temporal_Sup_R | 29 | 60 | -27 | 3 | -5.40 |
| Temporal_Inf_L | 56 | -42 | 0 | -42 | -5.26 |
| **lPD_post_ > lPD_pre_** |  |  |  |  |  |
| Parietal_Inf_L | 171 | -57 | -30 | 24 | 6.90 |
| Temporal_Sup_L |  | -54 | -15 | 9 | 5.09 |
| Postcentral_L |  | -55 | -18 | 24 | 5.03 |
| Frontal_Mid_R | 94 | 48 | 45 | -15 | 6.68 |
| Temporal_Mid_R | 86 | 57 | -18 | -12 | 6.56 |
| Frontal_Inf_Oper_L | 28 | -57 | 9 | 12 | 5.78 |
| Occipital_Mid_L | 40 | -27 | -75 | 27 | 5.52 |
| Cuneus_L | 39 | -18 | -63 | 21 | 5.39 |
| Precuneus_L |  | -12 | -57 | 15 | 4.38 |
| Postcentral_R | 28 | 66 | -15 | 33 | 5.30 |
| Cingulum_Ant_L | 43 | 0 | 51 | 9 | 3.74 |
| Frontal_Sup_Medial_L |  | 6 | 57 | 0 | 3.65 |
| Angular_L | 26 | -51 | -60 | 30 | 4.95 |
| Angular_R | 164 | 39 | -51 | 35 | 4.68 |
| Occipital_Sup_R | 36 | 24 | -63 | 42 | 4.58 |
| **lPD_post_ < lPD_pre_** |  |  |  |  |  |
| Frontal_Mid_L | 307 | -24 | 27 | 51 | -11.02 |
| Thalamus_L | 173 | -12 | -18 | 3 | -10.65 |
| Caudate_R | 32 | 12 | 12 | 21 | -7.35 |
| Cerebellum_8_R | 162 | 30 | -54 | -48 | -6.38 |
| Cerebellum_6_R |  | 30 | -39 | -30 | -4.51 |
| Caudate_L | 29 | -12 | 12 | 18 | -4.80 |
| Cerebellum_8_L | 31 | -24 | -51 | -54 | -4.74 |
| Cingulum_Ant_R | 51 | 0 | 27 | -3 | -4.40 |

*P* < 0.05, AlphaSim corrected; rPD, patients with right-side Vim thalamotomy; lPD, patients with left-side Vim thalamotomy.
