# Supplementary material for: Altered Activation in Cerebellum Contralateral to Unilateral Thalamotomy May Mediate Tremor Suppression in Parkinson’s Disease: A Short-Term Regional Homogeneity fMRI Study
Source: PLoS One. 2016 Jun 16;11(6):e0157562. doi: 10.1371/journal.pone.0157562 (PMC4910974; doi:10.1371/journal.pone.0157562)
Supplement: S2 Table — (DOCX) [file pone.0157562.s003.docx]

**ReHo differences between HS and PD patients in the pre-surgical condition**

| Region | No. of voxel | Peak MNI coordinate | | | Peak T intensity |
| --- | --- | --- | --- | --- | --- |
|  |  | x | y | z |  |
| **rPD_pre_ > HS** | | | | | |
| Cerebellum_6_L | 35 | -18 | -54 | -24 | 5.15 |
| Cerebellum_Crust1_R | 53 | 33 | -78 | -27 | 5.00 |
| Temporal_Mid_R | 36 | 54 | 9 | -24 | 4.81 |
| Cerebellum_6_R | 53 | 9 | -72 | -18 | 3.65 |
| Vermis_9 |  | 6 | -53 | -30 | 3.48 |
| Cerebellum_4_5_R |  | 9 | -50 | -12 | 2.89 |
| Cerebellum_Crust1_R | 24 | 42 | -54 | -33 | 3.56 |
| **rPD_pre_ < HS** |  |  |  |  |  |
| Temporal_Sup_R | 23 | 54 | -27 | 15 | -4.55 |
| Frontal_Mid_R | 25 | 33 | 6 | 57 | -3.68 |
| **lPD_pre_ > HS** | | | | | |
| Postcentral_R | 37 | 30 | -33 | 42 | 4.85 |
| Cerebellum_4_5_R | 68 | 15 | -42 | -21 | 4.52 |
| Frontal_Sup_Medial_L | 60 | -6 | 36 | 30 | 4.44 |
| Frontal_Sup_L |  | -12 | 33 | 39 | 3.90 |
| Cingulum_Ant_L |  | -12 | 42 | 15 | 3.88 |
| Cerebellum_6_R | 32 | 33 | -45 | -24 | 4.07 |
| **lPD_pre_ < HS** | | | | | |
| Frontal_Mid_R | 34 | 42 | 48 | 6 | -4.63 |
| Parietal_Sup_R | 32 | 18 | -72 | 54 | -4.31 |
| Frontal_Inf_R | 49 | 60 | 21 | 3 | -4.22 |
| Precentral_R |  | 54 | 9 | 12 | -3.57 |
| Occipital_Mid_L | 68 | -6 | -102 | 12 | -3.98 |
| Occipital_Sup_L |  | -18 | -93 | 18 | -3.22 |
| Occipital_Mid_R | 46 | 45 | -75 | 9 | -3.77 |
| Temporal_Mid_R |  | 51 | -69 | 3 | -3.59 |

*P* < 0.05, AlphaSim corrected; HS, healthy subjects; rPD, PD patients with right-side Vim thalamotomy; lPD, PD patients with left-side Vim thalamotomy.
