# Supplementary material for: Altered Activation in Cerebellum Contralateral to Unilateral Thalamotomy May Mediate Tremor Suppression in Parkinson’s Disease: A Short-Term Regional Homogeneity fMRI Study
Source: PLoS One. 2016 Jun 16;11(6):e0157562. doi: 10.1371/journal.pone.0157562 (PMC4910974; doi:10.1371/journal.pone.0157562)
Supplement: S3 Table — (DOCX) [file pone.0157562.s004.docx]

**ReHo differences between PD patients in the post-surgical condition and healthy subjects.**

| Region | Cluster size | Peak MNI coordinate | | | Peak T intensity |
| --- | --- | --- | --- | --- | --- |
|  |  | *x* | *y* | *z* |  |
| **rPD_post_ > HS** |  |  |  |  |  |
| Frontal_Sup_R | 52 | 9 | 51 | 30 | 4.70 |
| Frontal_Sup_R |  | 15 | 57 | 21 | 3.52 |
| Temporal_Mid_L | 45 | -45 | -57 | 21 | 4.40 |
| Frontal_Mid_L | 26 | -33 | 15 | 45 | 4.25 |
| Cingulum_Ant_R | 26 | 9 | 36 | 21 | 4.20 |
| Frontal_Sup_L | 23 | -12 | 54 | 24 | 3.99 |
| Frontal_Sup_L |  | -9 | 51 | 33 | 3.26 |
| Occipital_Sup_R | 24 | 18 | -93 | 24 | 3.94 |
| Temporal_Mid_R | 41 | 48 | -48 | 21 | 3.73 |
| Temporal_Sup_R |  | 50 | -54 | 21 | 3.59 |
| Parietal_Inf_R | 33 | 69 | -27 | 39 | 3.45 |
| **rPD_post_ < HS** |  |  |  |  |  |
| Frontal_Mid_R | 251 | 30 | 24 | 60 | -7.51 |
| Calcarine_L | 92 | -21 | -54 | 6 | -5.59 |
| Thalamus_R | 70 | 15 | -15 | 6 | -5.35 |
| Paracentral_R | 31 | 6 | -27 | 51 | -4.50 |
| Cerebellum_3_L | 39 | -6 | -42 | -21 | -3.52 |
| Hippocampus_L | 28 | -30 | -36 | 0 | -3.70 |
| **lPD_post_ > HS** |  |  |  |  |  |
| Fusiform_R | 28 | 30 | -72 | -18 | 5.62 |
| Frontal_Sup_R | 52 | 6 | 33 | 39 | 5.53 |
| Angular_R | 93 | 51 | -51 | 30 | 4.92 |
| Fusiform_L | 56 | -27 | -75 | -18 | 4.34 |
| Temporal_Sup_L | 54 | -42 | 12 | -6 | 4.33 |
| Insula_L |  | -39 | 3 | -9 | 3.14 |
| Cerebellum_4_5_L | 69 | -9 | -48 | -21 | 4.20 |
| Angular_L | 28 | -36 | -63 | 36 | 3.53 |
| Parietal_Inf_L |  | -42 | -54 | 39 | 3.29 |
| **lPD_post_ < HS** |  |  |  |  |  |
| Frontal_Mid_L | 227 | -27 | 27 | 51 | -7.92 |
| Thalamus_L | 98 | -12 | -18 | 3 | -5.54 |
| Cerebellum_8_R | 42 | 30 | -51 | -48 | -4.47 |

rPD, PD patients with right-side Vim thalamotomy; lPD, PD patients with left-side Vim thalamotomy.
