# Supplementary material for: Altered Activation in Cerebellum Contralateral to Unilateral Thalamotomy May Mediate Tremor Suppression in Parkinson’s Disease: A Short-Term Regional Homogeneity fMRI Study
Source: PLoS One. 2016 Jun 16;11(6):e0157562. doi: 10.1371/journal.pone.0157562 (PMC4910974; doi:10.1371/journal.pone.0157562)
Supplement: S4 Table — (DOCX) [file pone.0157562.s005.docx]

**Brain areas showing significant correlations between ReHo in PDpre and tremor score contralateral to surgical side.**

| Region | Cluster Size | Peak MNI coordinate | | | Peak R intensity |
| --- | --- | --- | --- | --- | --- |
|  |  | X | Y | Z |  |
| rPD |  |  |  |  |  |
| Parietal_Inf_L | 13 | -54 | -54 | 39 | 0.84 |
| Fusiform_L | 13 | -33 | -63 | -15 | 0.90 |
| Precuneus_L | 10 | -15 | -45 | 78 | 0.86 |
| Caudate_R | 9 | 12 | 9 | 6 | -0.84 |
| Cerebellum_4_5_L | 39 | -6 | -42 | -15 | 0.73 |
| lPD |  |  |  |  |  |
| Precuneus_R | 9 | 18 | -45 | 33 | -0.78 |
| Precuneus_L | 18 | -33 | -78 | 42 | 0.79 |
| Vermis_3 | 8 | 6 | -42 | -18 | 0.79 |

rPD, PD patients with right-side Vim thalamotomy; lPD, PD patients with left-side Vim thalamotomy.
